# T33. EARLY INTERVENTION FOR EARLY PSYCHOSIS IN FRANCE, MAPPING OF PROGRAMS

**DOI:** 10.1093/schbul/sby016.309

**Published:** 2018-04-01

**Authors:** Sophie Meunier-Cussac, Guy Gozlan, Laurent Lecardeur, Anne Duburcq, Laurène Courouve

**Affiliations:** 1 EMSI CHU Caen; 2 SAMSAH Prepsy; 3 Cemka

## Abstract

**Background:**

Early intervention programs (EIP) have been developed in many countries (United States, Europe, Canada, Australia) and are now widely considered effective in the treatment of early psychosis.

In France, current national policies in the field of mental health promote the development of early intervention but France has not yet met national standards of care for EIP.

A recent report from the London School of Economics (2016) comparing different European countries even mentioned the delay of France in this area, referring to only one EIP in the country.

However, different early intervention initiatives have emerged in France during the last decade without such information being centralized and therefore with no visibility on the current situation over the whole territory.

The aims of this study were to draw up a comprehensive inventory of existing or planned programs (in metropolitan France and in the overseas territories) in 2017 and to describe how they operate in 2016.

**Methods:**

This was a two-phase study; phase one was to identify and create an inventory of existing initiatives, and phase two was to describe and conduct an analysis of each initiative.

To be included, identified initiatives had to offer an early, intensive and multidisciplinary approach with at least 0.5 dedicated full-time equivalent staff. A secondary inclusion criterion concerned the out-patient setting of the initiative.

Inventory was achieved through many contacts across the country, among physicians/psychiatrists, healthcare facilities (hospitals, clinics, adolescent centers…) or administrative institutions (Health Regional Agencies…) which may either provide this kind of care or know of such initiatives.

An online declarative survey was administered between March and July 2017 to the identified psychiatrists with questions that covered administrative and clinical topics: structure of attachment, dedicated team, funding, targeted population, activity in 2016, partners of the program, difficulties encountered and prospects.

**Results:**

Between March and July 2017, 37 EIP for management of early psychosis were identified in France: 18 were operational, 8 were being established, and discussions were under way for the remaining 11.

The 18 identified operational programs were located throughout the country with a few regional disparities. All programs operated with multidisciplinary teams, including at least one psychiatrist and one nurse, and with a mean of 4.3 dedicated full-time equivalents healthcare workers (median: 3.7).

Most programs offered case management (12/18), with caseloads ranging from 4:1 to 22:1. The mean caseload was 10:1 (standard deviation 8:1).

All programs included 15 to 35 year-old early psychosis patients. Four programs also included patients at ultra-high risk for psychosis (UHR), while 4 others continued patient management during the chronic stages; 4 initiatives included all these stages of the disease.

Half of the programs had been existing for 2 to 5 years (50%); 89% were created less than 5 years ago.

The surveyed professionals described an increasing number of patients under their care.

**Discussion:**

Numerous projects and discussions appear to be under way (some programs should open very soon).

A real dynamic has been launched in France with an increasing focus and this evaluation will help to improve visibility of the identified programs and promote the development of new programs.

